# Discourse, ideas and power in global health policy networks: political attention for maternal and child health in the millennium development goal era

**DOI:** 10.1186/s12992-016-0157-9

**Published:** 2016-05-18

**Authors:** Lori McDougall

**Affiliations:** Department of Global Health and Development, London School of Hygiene and Tropical Medicine, 15-17 Tavistock Place, London, WC1H 9SH UK

**Keywords:** Millennium development goals, Sustainable development goals, Reproductive, Maternal, Newborn and child health, Policy networks, Discourse, Agenda-setting, Issue-framing

## Abstract

**Background:**

Maternal and child health issues have gained global political attention and resources in the past 10 years, due in part to their prominence on the Millennium Development Goal agenda and the use of evidence-based advocacy by policy networks. This paper identifies key factors for this achievement, and raises questions about prospective challenges for sustaining attention in the transition to the post-2015 Sustainable Development Goals, far broader in scope than the Millennium Development Goals.

**Methods:**

This paper relies on participant observation methods and document analysis to develop a case study of the behaviours of global maternal and child health advocacy networks during 2005–2015.

**Results:**

The development of coordinated networks of heterogeneous actors facilitated the rise in attention to maternal and child health during the past 10 years. The strategic use of epidemiological and economic evidence by these networks enabled policy attention and promoted network cohesion. The time-bound opportunity of reaching the 2015 Millennium Development Goals created a window of opportunity for joint action. As the new post-2015 goals emerge, networks seek to sustain attention by repositioning their framing of issues, network structures, and external alliances, including with networks that lay both inside and outside of the health domain.

**Conclusions:**

Issues rise on global policy agendas because of how ideas are constructed, portrayed and positioned by actors within given contexts. Policy networks play a critical role by uniting stakeholders to promote persuasive ideas about policy problems and solutions. The behaviours of networks in issue-framing, member-alignment, and strategic outreach can force open windows of opportunity for political attention -- or prevent them from closing.

## Background

The Millennium Development Goals (MDGs) have generated an increasing amount of reflection about how political attention has been shaped by these goals and how neglected issues could attract support in the future [1]. The MDGs, introduced in the early 2000s, include three main health goals, two of which focus on child and reproductive/maternal health. MDG4 calls for the reduction of under-five child mortality by two-thirds by 2015 against a 1990 baseline, and MDG5 calls for the reduction of maternal mortality by three-quarters during the same period, as well as universal access to reproductive health.

While neither the reproductive/maternal goal nor child health goal were reached by the 2015 target date [2], maternal and child mortality have each declined by half since 1990, and the global annual rate of reduction for child mortality doubled in the MDG era, from 1.8% during 1990–2000 to 3.9% during 2000–2015 [3]. Many have suggested that this improvement is linked to greater global political attention for maternal and child health during the MDG era.^1^ Indicators of such attention^2^ include policy statements and resource commitments, such as the 2010 launch of a “Global Strategy for Women’s and Children’s Health” by UN Secretary-General Ban Ki-moon, which attracted written “commitments” for implementation by more than 300 organisations, including 84 national governments. Estimates of official development assistance for maternal, newborn and child health (MNCH) rose from USD 2.67 billion in 2003 to USD 8.34 billion in 2012, despite a climate of declining overall aid contributions in recent years [4].

These events have taken place in a policy community marked by significant heterogeneity of actors, including those from government, donor agencies and foundations, multilateral agencies, academia, health professional associations, NGOs and private business. These actors are motivated by varying if inter-related interests, such as sexual and reproductive health, maternal, newborn, and/or child survival, and adolescent health. Further, they come from a wide range of epistemic traditions, resources, geographic networks and histories.

How did maternal and child health issues ascend on the policy agenda in the MDG era despite such heterogeneity of actor-groups? What are the challenges for sustaining attention when external conditions shift, as in the recent transition to the Sustainable Development Goals, the MDG’s post-2015 successor framework with a far-broader remit? And what might such challenges predict about the responses of such networks? These questions on political attention are explored in this paper through agenda-setting theory in political science, and discussed in the context of the rising power of public-private partnerships within the domain of global health governance and the practice of “global health diplomacy” [5, 6].

To explore these questions, this paper starts from Kingdon’s widely applied theory of “multiple streams” of policymaking. Rather than seeing policymaking a linear process of neatly demarcated stages, Kingdon suggests that agenda-setting, policy formulation, and implementation are part of an interactive process emerging from the confluence of three largely independent “streams” of “problems”, “policies” and “politics”, each with its own highly dynamic character [7]. Kingdon refers to “problems” as those emerging from a process of competition among advocacy actors in which social conditions are successfully portrayed or “framed” as urgent and amenable to public action, thereby attracting political attention. “Policies” refers to the various ideas and solutions proposed by policy communities to address problems as agreed. “Politics” is the larger environment in which this competition plays out. This stream includes elements such as political “mood”, the inclinations of governing regimes, and prevailing social trends. Kingdon sees these streams coming together at certain moments in time through the successful manipulation by individual policy “entrepreneurs”. This process levers open “windows of opportunity” through which advocacy actors can successfully pursue their goals.

Kingdon’s theory of policymaking has been influential in drawing attention to agenda-setting as the outcome of highly dynamic interactions between ideas, actors, and context. In this tradition, Walt and Gilson’s “triangle” framework [8] for conducting health policy research is valuable in drawing attention to how actors, context and processes interact with policy content in the shaping and reshaping of that content. This triangle highlights power relations in such arenas, and as such is particularly relevant for studying the ideas, discourse, and behaviours of actor-networks in pursuit of certain advocacy goals – a key concern of this paper.

To help identify, organise and analyse this case study on agenda-setting for maternal and child health, this paper applies Shiffman and Smith’s 2007 framework of policy prioritisation [9]. Grounded in Kingdon’s concept of multiple streams, as well as Walt and Gilson’s dynamic approach to health policy analysis, Shiffman and Smith highlight four main areas – i) ideas; ii) actor power; iii) political context; and iv) issue characteristics – that combine to explain agenda-setting effects. This paper will focus on the first three factors as the most relevant to the case of the MNCH community, with specific discussion on each.

In setting out this framework, Shiffman and Smith call particular attention to the agency of actors and their “guiding institutions”, such as advocacy networks – from the ideas that they select, to the leaders and guiding institutions that they choose, to the indicators they prioritise to demonstrate severity of their issues and the credibility of their proposed solutions. How power is mobilised, asserted and used by actors is intrinsic to these processes, and is central to this study.

While Shiffman and Smith do not assign causal weight to these four broad categories of factors within their framework, this study on maternal and child health will pay particular attention to the influence of actor-power through the formation of networks, leadership, and institutional development. A key question in this study is how actor cohesion developed in spite of heterogeneity. As noted, this diversity among actors is multi-dimensional, spanning different geographic locations and interests, constituencies (e.g., private sector, health professional, donors, NGOs, etc.), and professional training (e.g., life sciences, economics, international relations, management, finance, sociology, law, etc.).

Following Smith and Smith, actors are understood in this paper not only as individuals and organisations with their own knowledge, attitudes, practices and behaviours, but as networks of actors, including “networks of networks” such as The Partnership for Maternal, Newborn & Child Health (PMNCH), formed in 2005 to unite three previously separate global networks on maternal health, newborn health and child survival.

Keck and Sikkink characterise networks by their “voluntary, reciprocal and horizontal patterns of communication and exchange” [10]. Such networks may be closely connected or tightly structured, but as institutions, they are distinguished by their capacity to participate in collective action [11]. Kahler [12] contrasts this concept of “actor-based” networks with more static, “structure-based “networks, originally developed in economics and sociology literature of the 1960s and 1970s [13, 14] as relatively unyielding, insensitive edifices that themselves shape the behaviour of its constituent members to produce desired effects. This case demonstrates that the capacity of actors to shape their structures has enabled the timely and effective negotiation of goals, strategies and approaches, which has in turn helped force open policy windows and facilitate access to policymakers. In this way, the structure of the network itself is a determinant of success of advocacy goals, and participants continually retool that structure in line with shifting opportunities and political conditions.

How ideas are negotiated and portrayed to both internal audiences (e.g., network members and allies) and external audiences (e.g., policymakers, media and others influencers) are key aspects of the Shiffman and Smith framework. Benford and Snow [15] see the framing of problems and policy solutions as outcomes of contested process among actors. Given the wide array of social issues and conditions that policymakers are confronted with every day, advocacy actors vie with each other to construct effective “causal narratives” [16] and storylines to manipulate how issues are perceived and what policymakers believe can be done about such “problems” [15]. This view assumes that empirical evidence alone is insufficient to motivate action, especially when certain issues – such as maternal, newborn and child mortality – have been regarded habitually as longstanding issues with complex and expensive policy solutions. To counter this, actors must negotiate among themselves to agree and communicate collective action “frames” that cast a new, actionable light on their issues, and promote network coherence by assisting members in locating, perceiving, identifying and labelling their experiences [17].

In Gitlin’s definition, frames are as “the persistent patterns of cognition, interpretation, and presentation of selection, emphasis and exclusion, by which symbol-makers routinely organise discourse” [18]. McInnes et al. identify five frames most frequently used in global health: biomedicine (i.e., evidence-based medicine), economics, security, development, and human rights [19]. The positivist approach of “evidence-based medicine” describes a world in which material outcomes can be shaped by applying epidemiological and biostatistical analysis and solutions to the policymaking process. In the field of public health, the evidence-based medicine frame is often combined with an economic frame to assert significant returns on investment by scaling up coverage of “packages” of biomedical interventions, such as contraceptives, vaccines, and skilled care at birth, supported by improved health delivery systems. Through this equally positivist frame, the emphasis on cost-benefit analysis and return on investment dates arises from a neoliberalist concept of development popularized in the 1980s and still widespread today. Development problems, such as maternal and child mortality, are thus seen as amenable to market solutions, such as more money, more information, and greater operational efficiency. In comparison, structural barriers rooted in inequalities of place, race or class often have a less visible explanatory role in this narrative.

Evidence-based framing in public health has risen significantly in the past decade as part of a wider thrust towards evidence-based medicine and clinical practice. For example, evidence-based framing has been adopted by the maternal health community in a bid to “professionalize” its advocacy through biomedical and economic framing, thus reducing reliance on moral arguments in swaying the attention of political leaders [20, 21]. For many in public health, the maxim of “What gets measured, gets done” remains a literal statement of truth, emphasizing the power of quantifiable measurements to attract attention and motivate action. As Foucault observed, such discourses are practices that systematically form the objects of which they speak, investing power and authority in the projection of an objective measurable “truth” [22]. Indeed, problems and solutions can be oversimplified when framed and promoted by those in positions of influence, taking on the status of “master narratives” that in themselves shut down debate and limit the scope of ideas for change [23].

The emphasis on quantitative targets and measurements in the MDG framework has clearly influenced the discourse of actor-networks, as well as the policy audiences they have sought to influence. This can be explained because frames are most likely to be accepted by policymakers when they resonate with public understandings and provide new ways of talking about and understanding issues [24]. Ideas, therefore, therefore, must be considered central to the relationship between evidence and public health, suggesting a more complex relationship than the oft-depicted linear, causal link between science and policymaking.

At the same time as the MDG discourse, the reorientation of development practice to foreign policy concerns has also had a structuring effect on how multi-stakeholder networks have framed their issues, aligned their members and built their alliances. This includes a tactical recognition of “high politics” [25] as a driver of attention to issues deemed vital to state survival, including security and economics -- and a corresponding influence in how issues are agreed and portrayed.

The conceptual shift from the MDGs to the SDGs – from a neoliberal market-oriented view of development to a “people-centred” view of development -- raises questions, therefore, about how actor-networks and their ideas respond to such conceptual and contextual shifts in a bid to retain power, resources and attention.

This question summarises the central concern of this paper with the agenda-setting process. Against a background of the MDGs and new forms of global health governance, how was attention for maternal and child health achieved in an arena populated by disparate organisations with different experiences, different measurements, and different causal stories? Who set the terms of the “frame development” process, what history did that build on, and what trade-offs were made during the process of consensus-building?

To succeed in the SDG era, networks will be challenged to behave in ways that frame health not as an isolated technical domain, but as a determinant, outcome and indicator of sustainable development itself [26]. What do those conditions suggest to networks in reshaping norms, behaviours, and structures to sustain attention for maternal and child health issues?

## Methods

This study approaches agenda-setting from a social constructivist perspective [10], taking a detailed case study approach to understanding how and why ideas are constructed by communities of actors, and how those ideas influence power and policy.

Case studies enable researchers to analyse “real life” processes through a combination of observatory, textual and process-tracing methods, revealing underlying information that can help explain “how” and “why” such processes occur [27].

Given the interest of this paper in how networks use frames to achieve political attention, a participant-observation approach was used to identify the process by which key ideas and frames are negotiated, agreed and contested. Access to this network discourse was privileged by the author’s employment in the secretariat of The Partnership for Maternal, Newborn & Child Health (PMNCH), beginning from PMNCH’s formation in 2005, through to the events described in this paper. PMNCH is a “network of networks” where information from different member-organizations comes together. From 2005 to 2015, PMNCH grew from less than 100 member-organizations to 725 member-organizations across eight constituencies: national governments, donors and foundations, NGOs, multilateral agencies, health care professional associations, private business, youth and adolescents, and academia. Potential problems of being an employee and a researcher at the same time were avoided as the main subjects of the study were constituent organizations rather than the secretariat itself.

Data was collected on a regular basis through direct observation of global, regional and national meetings, verbal and written exchanges among network members and informal discussions. For example, public speeches at assemblies and conferences were analysed to identify changes in mainstream policy discourse. Ethical approval for this study was granted by the London School of Hygiene and Tropical Medicine as part of the author’s doctoral dissertation research. Within the wider maternal, newborn and child health community, the author’s research interests were widely known and referred to on the secretariat biography page of the PMNCH website.

To address self-bias inherent to a participatory approach [11], a review of approximately 200 published and unpublished documents was also undertaken. These documents were produced by several different epistemic and professional groups, including those with specific reproductive, maternal, newborn, child and adolescent health interests and expertise. These documents were assessed to triangulate narratives on the historical development of the global maternal and child health community, including perceived milestones, successes, challenges and risks in the current period of transition from the MDGs to the SDGs. Most of these documents were identified through the PMNCH web archive covering the years from 2005 to 2015, which reported news and reports from many constituent organizations, including web links to their major reports, press releases and speeches. Additional documents that predated the PMNCH archive were found through references in the reviewed documents. The evolution of framings could be mapped through these publicly available documents, including through reported speeches, policy papers, strategic plans, annual work plans, and reports. Furthermore, a number of peer-reviewed papers, co-published by practitioners and political actors in academic journals, were included in the document analysis due to their normative nature reflecting the framing of the policy discourse.

The method of analysing the data from participant observation and the documents was based on open-ended coding of relevant conceptual labels and themes and their subsequent merging into broader categories that eventually coincided with the observation scheme. A timeline was developed to identify key moments in the development of frames, products, and external events relevant to network structure and operation (Table 1).

**Table 1:** Ideas, actor-groups, and political context in relation to the development of a global MNCH community, 2005–2015

2005–2010			
Ideas	Political context governments	Political context multilateral	Political context civil society
*MDGs 4 & 5 present opportunity to create a joint “MNCH” identity for greater mutual impact*	2006: African Union (AU) announces Maputo plan on sexual and reproductive health to accelerate MDG results	2005: World Health Report, *Make Every Mother and Child Count*, promotes continuum of care, calls for enhanced progress in reducing mortality	2005: PMNCH formed as “super-network” of maternal, newborn and child groups to advocate for joint achievement of MDGs 4 and 5
*“Continuum of care” created as operational framework for integrating maternal and child health service delivery*	2006: Norwegian PM Stoltenberg convenes new Global Business Plan for MDGs 4 & 5; 2007, announces $1b for MNCH, launches head of state network on RMNCH	2007: MDG5b created on reproductive health	2005: Countdown to 2015 progress report grows out of *Lancet* special reports on child and newborn health
*Evidence-based advocacy, based on epidemiological and economic measurements, adopted by wide range of MNCH actors. Rights/moral-based messaging declines.*	2008: Inter-Parliamentary Union co-hosts global meeting with Countdown to 2015 on RMNCH	2009: UN Secretary-General Ban Ki-moon identifies maternal and child survival as priority for action; calls for development of global plan of action	2006: Lancet produces evidence series on maternal health, anchored by new global epidemiological analysis
*Norms and beliefs articulated through the Global Strategy for Women’s and Children’s Health (2010)*	2009: AU launches national CARMMA campaigns to advance Maputo plan	2009: High-Level Task Force on Innovative Financing influences first-ever costing of gap in reaching MDGs 4 and 5	2007: Women Deliver holds first global conference in London, uniting advocates and marking pivot to evidence-based advocacy
*Multi-stakeholder “partnership” narrative asserted to promote delivery of Global Strategy and influence of MNCH policy community in global health arena*	2010: G8 pledges US$5b for MNCH at Muskoka summit in Canada	2010: *Global Strategy for Women’s and Children’s Health* led by UN Secretary-General Ban Ki-moon attracts US$40b in pledges through a new partnership platform, Every Woman Every Child	2009: MNCH Consensus agreed among broad range of UN, civil society, donor, and health professional partners: first-ever technical and financial consensus
	2010: AU heads of state hold summit on RMNCH		
2011–2015			
Ideas	Political context: governments	Political context: multilateral	Political context: civil society
*Accountability discourse takes hold in MNCH community to measure progress towards MDGs and Global Strategy – technical orientation cedes gradually to rights-based measures of accountability as SDGs take shape (2011–2015)*	2011–12: Parliaments intensify support for MNCH through Inter-Parliamentary Union resolution and Pan-African Parliament resolutions	2011: Commission on Information and Accountability for Women and Children, chaired by leaders of Canada and Tanzania, sets out goals and targets based on the Global Strategy. Calls for creation of an “independent Expert Review Group” to track progress, reporting to UN Secretary-General	2011: PMNCH opens private sector constituency, recognising contributions in innovation and efficiency
*Increasing complexity of MNCH architecture underlines need for global health governance reforms*	2012: DFID, BMGF and UNFPA convene London Family Planning Summit, raising $2.4b in pledges; FP2020 created to support and track progress, linked to the Global Strategy	2013: Global Investment Framework for Women’s and Children’s Health launched in conjunction with Lancet Commission on Investing in Health	2012: First annual report of independent Expert Review Group emphasises need for stronger global health governance, national data, human rights and participation
*Rising economic power of LMICs and stagnating donor aid prompts greater shared concern with national leadership and financing*	2014: Open Working Group report on post-2015 SDGs emphasises integrated health and development goals, based on sustainability and human rights, including adolescent and reproductive health	2014: Global Financing Facility for Every Woman Every Child created to harmonise aid and leverage domestic funds	2014: PMNCH publishes *Success Factors* study to advocate for stronger links between health outcomes and social and economic determinants in post-2015 era
*Emergence of integrated SDG framework highlights focus on social, political, economic and environmental determinants of health*	2015: MDGs end, SDGs launched	2015: Updated *Global Strategy on Women’s, Children’s and Adolescents’ Health* developed in support of SDG agenda	2015: “Citizen Hearings” on women’s and children’s health led by NGO coalitions at sub-national, national, and global levels to demand greater accountability

## Results and discussion

The interconnected needs of women and their babies have been long recognised. Declarations of the 1990 World Summit for Children and the 1994 International Conference on Population and Development both articulated this concept. This was further elaborated by the World Health Organisation in 1996 through its “mother-baby” technical guidelines [28]. Even so, the proposed interventions, institutional leadership, historical development and analytical frames associated with each cause were sufficiently different as to engender largely separate advocacy movements, with a fair degree of resource-competition between them [29–32].

The advent of the MDGs in the early 2000s set the stage for a shared advocacy approach. The MDGs allocated two of its eight goals for child and maternal health: MDG 4 (reduce under-five child mortality by two-thirds by 2015 from 1990 baseline) and MDG 5 (reduce maternal mortality by three-quarters by 2015 from a 1990 baseline). Reproductive health, sidelined from the MDG agenda, was eventually added as a sub-goal to MDG 5 in 2008. The MDGs, though widely criticised for promoting a depoliticised, decontextualised view of development [29, 33, 34], began to gain support for their agenda-setting power, drawing high-level attention and consensus around a simple, easily communicated set of goals.

Since maternal and child health advocates were positioned by the MDGs as equal partners in this high-level political project, it seemed strategic to join forces with each other to maximise policy attention and results by presenting a combined burden [21, 35]. However, to do so, it was critical to align conceptual and organisational approaches to serve the interests of a wide range of heterogeneous partners. In the mid-2000s, this gave rise to the creation of the “continuum of care” framework as an inclusive operational approach. This framework was popularised by a new joint institutional base founded to promote a collective identity for advocacy, The Partnership for Maternal, Newborn & Child Health, as discussed below.

Using Shiffman and Smith’s framework of ideas, actors and political context, this paper will explore each of these concepts in turn to explore the case of the MNCH.

### Ideas: MNCH frames and evidence-based discourse

The “MNCH continuum of care” conceptual framework, promulgated through a series of high-profile publications by key policy actors in 2005 [35, 36], expanded the concept of the mother-baby dyad to incorporate determinants and outcomes of healthy pregnancies and safe deliveries, including strong health systems. It proposed the seamless delivery of health interventions through an integrated view of time (from pre-pregnancy to pregnancy, delivery, and early childhood), as well as space (from community level up to facility level). The assumed logic of the framework was better care through, in part, greater resource efficiency. Investments in one area would benefit others, reducing pressure on maternal, newborn and child health advocates to compete for resources and attention [35–37] as a PMNCH ‘fact’ sheet commented:

*The Continuum of Care recognises that safe childbirth is critical to the health of both the woman and the newborn child—and that a healthy start in life is an essential step towards a sound childhood and a productive life* [38].

Early descriptions of the continuum of care positioned rights at the centre of the frame, acknowledging the politics that surrounded it. Rejecting the vertical approaches that had dominated global health in the 1990s and early 2000s, including through the rapid rise of new global health partnerships dedicated to particular diseases and technical interventions [39, 40], proponents such as the authors of the 2005 World Health Report, *Make Every Mother and Child Count*, called for a broader health systems approach rooted in equity concerns:

*Maternal, newborn and child health cannot be reduced to a set of programmes to be delivered to a target population. Rather, mothers and children must be in a position to claim a set of entitlements as their right. This implies an adjustment of macro-level health policies and resource mobilisation, at country level and internationally* [36].

While the continuum of care concept was appropriately large and ambitious, at a practical level, it remained difficult to quell longstanding tensions related to a large range of issues embedded within the framework. These included broader issues relevant to all network actors, such as attention to community-based vs professional and facility approaches or health system investments vs disease-specific investments, but also tensions among communities, such as how sexual and reproductive health and rights would be recognised and prioritised within an integrated “MNCH” approach that focused largely on the supply and monitoring of biomedical interventions and clinical services.

Such debates had run deep in the era of the “safe motherhood movement” in the late 1980s and 1990s [9, 20]. The child health community, too, had also experienced tensions about how best to set a course for progress, including the role of community-based care versus facility care. By the early 2000s, there was a general concern of slowing progress in both the maternal and child community. Leading scientists, public health specialists and journalists affiliated with the child survival community, for instance, expressed public concern that the momentum in child mortality reduction achieved in the 1980s and 1990s during the “golden years” of Jim Grant’s leadership at UNICEF had waned dramatically [41].

Grant’s focus on scaling up coverage of key interventions such as treatment of diarrhoea and pneumonia, immunisation, protection from malaria, and attention to nutrition, had achieved important gains in survival during his tenure. However, Grant’s successor, Carol Bellamy (1995–2005), shifted the focus from intervention coverage to a broader agenda, uniting health with a range of related concerns, including girls’ education and child protection. Bellamy argued that the child survival agenda needed to adapt to changing times, moving “beyond survival” to focus on human rights, reflecting the conclusion of the “World Fit for Children” agenda of a major UN session on children in 2002 [42]. Tensions surfaced in a Lancet editorial in 2005, in which editor Richard Horton charged Bellamy with dropping the ball on the “essential” health needs of children:

*A preoccupation with rights ignores the fact that children will have no opportunity for development at all unless they survive. The language of rights means little to a child stillborn, an infant dying in pain from pneumonia, or a child desiccated by famine. The most fundamental right of all is the right to survive* [43].

Horton was not a neutral party: In 2003, the Lancet had published a highly influential series on child survival by the “Bellagio study group”, whose members included senior epidemiologists and academicians of global repute such as Bob Black and Jennifer Bryce of the US, Cesar Victora of Brazil, Zulfiqar Bhutta of Pakistan and Hassan Mshinda of Tanzania. Together, the Bellagio group put forward a powerful case for renewed global attention to child survival, demonstrating through statistical, economic and policy analysis where and why problems lay and how they could be addressed [41].

The message of the Bellagio group was clear: To generate greater investment and political will, monitoring and reporting on progress was crucial. Governments, donors, the UN and other policy actors could be better held to account through high quality data and analysis on health indicators; interventions, including inequities in coverage; resource flows; and health system policies and legislation.

Given the combined status and reputation of the Lancet and Bellagio authors, their call to action held considerable sway over the global child health community, as well as within their respective geographic networks. Given the overall normative thrust of the MDGs towards technical and managerial solutions to development and health as reported in numerous academic studies [1, 33, 34, 44], the emphasis of the Bellagio group on quantifiable scientific evidence was widely accepted as an important contribution to monitoring progress on the MDGs. This example of evidence-based advocacy in public health was a sign of the times, echoed in the metrics of the MDGs as well as in the neoliberal economics in many donor countries at the time [19]. The concept of “selective primary health care” itself was a re-interpretion of the idealistic 1978 Alma Ata agenda, now focusing on “practical”, time-bound and measurable health interventions [21].

The emerging “evidence-based” advocacy approach of the MNCH community was cemented in the launch of the “Delhi Declaration on Maternal, Newborn and Child Health” in April 2005, which announced the formation of a new combined platform for action, the “Partnership for Maternal, Newborn & Child Health” [45]. Sexual and reproductive health, while clearly evident in the concept of the continuum of care, was mentioned in the Delhi Declaration, if lacking in emphasis. The word “equity” and “rights” appeared just one time each in the text of the Declaration, compared with eight combined references for “coverage” and “resources” [45]. Reproductive health organisations, perhaps preoccupied in part with the struggle to gain belated inclusion of reproductive health within the MDG framework [44], appeared content to let maternal and child advocates get on with their work in forming an “MNCH” super-network. Indeed, PMNCH seemed to be understood by the reproductive health community as a creature of the MDGs, reflecting its techno-managerial framing, and therefore, perhaps of limited long-term strategic value in the struggle to recognise rights [30].

Within the MNCH community, issues like abortion rights and sexual health education – controversial to some members of PMNCH, including conservative governments and faith-based NGOs – were often omitted or downplayed in public messaging. The adoption of “neutral” scientific framing and discourse was instrumental in reducing scope for friction among the many sub-communities of the continuum of care.

Indeed, Countdown to 2015, a scientific collaboration growing out of the Bellagio Group to track the progress of MDGs 4 and 5, did not include abortion-related indicators in its flagship reports until 2014 [46], when it published a box about preventing unsafe abortion and the number of Countdown countries with legislation permitting abortion. Although well regarded for its pioneering and innovative approaches to tracking health inequities, Countdown's reporting on trends in national coverage of key health and nutrition interventions is the dominant focus of reporting, complemented by tracking of financial and policy indicators. In this sense, Countdown to 2015 reflects the dominant discourse of the MNCH community over the past 10 years, legitimising and disseminating evidence-based framing through the “gold standard” authority of its work.

This discourse is echoed throughout key policy documents in the MNCH community during the MDG era [45, 47]. This was clearly reflected in the original *Global Strategy for Women’s and Children’s Health* (2010–2015), launched by the UN Secretary-General to accelerate progress on the MDGs:

*Together we must make a decisive move, now, to improve the health of women and children around the world. We know what works. We have achieved excellent progress in a short time in some countries. The answers lie in building our collective resolve to ensure universal access to essential health services and proven, life-saving interventions as we work to strengthen health systems. … Often the solutions are very simple – clean water, exclusive breastfeeding, nutrition, and education on how to prevent poor health are only a few examples. … With the right policies, adequate and fairly distributed funding, and a relentless resolve to deliver to those who need it most – we can and will make a life-changing difference for current and future generations* [47].

In this view, mortality is seen as the dominant problem, and the Global Strategy set out a prescription for more money, better policy and programme support, greater efficiency through harmonisation of actors, better data and information, and above all, “more resolve” by all stakeholders.

Even attention to health systems, which has grown significantly from the mid-2000s in response to the proliferation of global health initiatives and vertical initiatives [40], is framed in managerial terms, i.e., that weak systems represent threats to MDG progress and “bottlenecks” to organisational objectives [48]. Health systems are projected mainly as technical delivery channels of commodities, interventions and workers, not as social institutions shaped, and shaped by, the interaction of people, policies and services. Demand-side policies and community voice in the context of political reform were nearly entirely absent from the 2010–2015 *Global Strategy* – now corrected in its successor strategy, the *Global Strategy for Women’s, Children’s and Adolescents’ Health*, launched in September 2015 to align with the equity and rights-focused Sustainable Development Goals for 2016–2030.

In summary, the use of quantitative data and the deliberate scientific framing of maternal and child health by network leaders, such as by the leaders of the Countdown movement and PMNCH, satisfies two key conditions required for political attention: severity of the issue, and the feasibility of solutions proposed [15, 49].

Shiffman and Smith’s framework of ideas, actor power, political context and issue characteristics treats these categories in a dynamic fashion, recognising the mutual influence of these domains. Frames in this study are seen in a similar fashion – they are agreed by actor-networks within a certain political context, whose behaviours in turn are shaped by their use of such frames.

### Actor power: MNCH advocacy networks in the global health arena

The formation of these MNCH networks and their political use of frames occurred in the midst of a major shift in global health governance, in which the concept of “international health” among sovereign states was replaced by the idea of “global health” characterised by the interdependence of nations and sectors through globalisation processes [5, 6, 39, 50].

A new cohort of health actors claimed power and contested the traditional governance arrangements of international health. From the early 2000s, and spurred in part by the MDGs, came a wave of 100-odd new private-public “global health initiatives” [51]. Issues included HIV/AIDS (e.g., Global Fund to Fight AIDS, Tuberculosis and Malaria in 2002; PEPFAR in 2003), TB (Stop TB, 2001), child health (GAVI in 2000, Child Survival Partnership in 2004), maternal health (Partnership for Safe Motherhood and Newborn Health in 2004, emerging out of the longstanding Inter-Agency Group for Safe Motherhood), and newborn health (Healthy Newborn Partnership in 2000) among many others.

By the mid-2000s, global health discourse had shifted to the need for coordination, harmonisation, and accountability among all of these actors. This discourse introduced concepts of partnerships for “aid effectiveness” and intensified discussion on global health reform and the need for “global solidarity”, given increasing cross-border, cross-constituency issues such as the rising burden of non-communicable diseases, climate change and other environmental crises, pandemics, population migration and information flow [51].

This set the scene for the birth of The Partnership for Maternal, Newborn & Child Health in 2005, uniting three previously separate partnerships: the Partnership for Safe Motherhood and Newborn Health, the Healthy Newborn Partnership and the Child Survival Partnership [52]. Members were divided into different “constituencies”, representing institutional affiliations (governments, NGOs, academia, multilaterals, health professional associations, donors/foundations, and later private business).

Since leaders from all three networks had agreed to the continuum of care framing, tensions between the networks focused less on ideational differences than realpolitik, such as which member would host the new secretariat and enlarge its reputation accordingly. The PMNCH board was deliberately large to accommodate representative seats from multiple constituencies and communities, removing a potential source of tension.

In PMNCH, maternal health networks spotted a fresh opportunity for “evidence-based advocacy”, turning the page on a complex past of internal dispute and slow progress as other studies suggested [9, 20, 53]. Child health networks, spurred to action through the emerging Countdown movement and the “rhetorical power” of MDG 4 [42], saw an opportunity to expand their support base; and newborn advocates, still few in number, saw an opportunity for growth through alignment, justified by the continuum of care concept. To most concerned, including those concerned with better aid coordination, PMNCH seemed like a win-win.

In its 10-year history, PMNCH has developed into what Shiffman and Smith would describe as a “guiding institution” [9] of 725 member-organisations, assuming a leadership role in producing consensus among network members.

A historic analysis of PMNCH board documents (2005–2015) published on its web site [54] finds extensive evidence of a culture of diplomacy and member cooperation, regardless of underlying tensions between constituencies and members, including on issues such as abortion and sexuality education among adolescents. Debate on such “red flag” issues are largely absent from the official record, suggesting they are not raised (or not reported).

When evidently sensitive issues do appear on the agenda, such as those related to financial resources or governance arrangements, the the public discourse can be highly diplomatic, with official notes referring only obliquely to issues of power and transformation. For example, the note of the PMNCH December 2014 strategic board retreat quoted its board chair in relation to the clear challenges that lie ahead in transitioning from the MDGs to the SDGs, and the need for negotiation among actors in this process, but framed such political considerations as almost "technical" in nature:

*Graça Machel noted the considerable challenges of the new agenda for women and children, and stressed the priority of ‘leaving no one behind’. The magnitude of the task ahead will require scaling up activities significantly and negotiating the inclusion of robust accountability mechanisms that will track progress for women, children’s and adolescents’ health in the years to come* [55].

Formal governance meetings and related written reports often have little direct focus on challenging dynamics. For instance, the note from the December 2014 PMNCH board meeting made little reference to divided opinion about the Global Financing Facility for Every Women Every Child, which was the subject of a public consultation coordinated by PMNCH at the time [56].

Public suppression of conflict is observed to be a tactical behaviour in the MNCH community, borne out of the political need to achieve coherence among institutions, epistemologies, and professional backgrounds within the network. As such, the “neutral” scientific framing adopted by the community can also be understood as a tactic for suppressing conflict and facilitating coherence within the network, as well as a powerful external frame in the “high politics” arena.

With internal tensions carefully managed within its governance structures, the MNCH community has been successful overall in presenting a cohesive public face – a key “actor power” attribute of the Shiffman and Smith explanatory framework. An example of this actor coherence – and its contribution to attracting political attention – is the production of the *Global Strategy* by PMNCH members in 2010, and its contribution to the mobilisation of an estimated USD60 billion in related financial resources through more than 400 written commitments to the strategy [55].

Such cooperation, however, does not suggest lack of tensions between sub-communities – reproductive, maternal, newborn and child. In 2013–14, the newborn health community developed a high-profile action plan, Every Newborn, which was supported by a resolution among 192 member-states of the World Health Assembly in 2014 [57]. Some maternal advocates had tried to persuade the newborn advocates to slow down and develop a fully integrated maternal and newborn health plan, concerned that a newborn-focused plan would distort attention and undermine longer-term goals of maternal-newborn integration. Reasons for friction were explained in the Lancet by former PMNCH co-chair Ann Starrs soon after the Every Newborn launch:

*The maternal and newborn health communities must move beyond the lingering tensions that limit full cooperation and acceptance of each other's priorities. On the maternal health side, this wariness reflects a concern that embracing the newborn baby would inhibit efforts to address reproductive health and to anchor programmes and policies in a rights framework. On the newborn health side, there are concerns that embracing the full maternal health agenda might slow the momentum of the Every Newborn Action Plan and compromise its achievements* [32].

### Political context: MDG to SDGs

The MNCH case discussed in this paper justifies this important focus on actor behaviour, as seen through the manipulation of frames and strategic development of multi-constituency networks (Table 1). However, the efficacy of these behaviours has been determined, in part, by two key external factors: the MDGs and the expanding power of non-state actors within the global health governance arena more generally.

The MDG 4 and 5 “dyad” has provided a focus and justification for ideational alignment between policy sub-communities (i.e., based on the continuum of care concept, communicated through the evidence-based framing of the Global Strategy). This conceptual agreement has, in turn, provided a catalyst for structural and behavioural alignment between sub-communities (e.g., the development of PMNCH and the creation of Every Woman Every Child and related campaigns, such as Every Newborn).

As a result of this alignment, stakeholders have been able to advance on two fronts. One is the acceleration of progress towards the 2015 MDG goals. The second is the shaping of the new post-2015 SDG goals and their delivery mechanisms. Several goals in the SDGs are conditioned by the need for greater progress on women’s and children’s health, including those on education, gender, water and sanitation and others. This brings the concern of health into the centre of the SDGs, away from the margins, where many fear it will lose attention as just one among 17 competing goals [26, 58]. At the same time, the operational model of the Global Strategy, through its Every Woman Every Child private-public partnership platform for leveraging resources and tracking results, is also promoted as an innovative delivery model for the SDGs themselves.

These examples suggest at least a partially reciprocal relationship between political context, actors, and ideas. Political context and policy windows, while often far beyond the domain of advocates, can also be influenced to some extent through collective action to secure political attention.

But what happens when political conditions change, as in the current shift from the MDGs to the SDGs? Some advocates fear, for instance, that a much-expanded framework will tilt political attention away from health, no matter how effectively such issues have been “embedded” in several goals. Can such risks be mitigated through the realignment of ideas and behaviours, or is context simply more powerful?

Early signs of adaption are occurring in the MNCH network in relation to ideas and frames, as well as network behaviour, structure and leadership. For example, the updated Global Strategy for the SDG era reflects updated technical evidence, but its chief characteristics are the broadening of its scope of concerns and the universality of its application. The 2010 edition had a tight focus on interventions related to maternal and child mortality in low-income countries, with far less prominent attention to social and economic determinants of health, as well as adolescent and reproductive health, infectious disease, and nutrition. The 2015 document, by contrast, adopts a “beyond mortality” lens, proposing a rights-based vision of “survive, thrive and transform”. Boundaries of concern are therefore greatly expanded, including to fragile and humanitarian settings [59].

The SDG framework is similarly ambitious, developed through an extensive country-led consultation process during 2012–2015. The resulting framework speaks to its democratic process, and includes 17 goals, 169 targets and an even greater number of performance indicators. By comparison, the MDG framework had just 18 targets and 48 indicators, leading to focused political attention -- as well as extensive backlash among those who saw the MDGs as far too utilitarian to produce meaningful social change.

The development of the updated Global Strategy has followed the SDG themes closely, sending key messages about the primacy of national leadership, equity, and mutual accountability of stakeholders. Virtually no dissent has been heard about the primacy of adolescents and youth in the new Global Strategy, including greater attention to related issues such as early and child marriage, family planning, and adolescent access to health services. This stems from several reasons. First, shifting demographic patterns and rising youth populations in many countries with growing economic power, including those in Africa, have triggered new recognition of the importance of sexual and reproductive health policies. For instance, the African Union’s CARMMA campaign, launched in 2009 to support continental policies on sexual and reproductive health, is now active in 44 countries.

Second, at a global level, the long-standing struggle to secure attention to reproductive health, resulting in the belated achievement of MDG 5b in 2007, cemented strong networks for action that have continued to yield results. The Women Deliver global conferences, beginning in 2007, have brought advocates together from all regions. Donors such as the Bill & Melinda Gates Foundation have partnered with the UN and countries such as the UK, India, Ethiopia and others to push family planning issues ahead, creating the “FP2020” global network in 2013.

Together, reproductive and adolescent health have pushed the MNCH community to refresh its discourse, members, and strategies, creating what is known as the “RMNCH” community (or “RMNCH + A”, including adolescents). This emergence been marked by important new commitments. At a global level, Overseas Development Assistance for reproductive health has increased significantly in recent years, from USD3.6b to USD4.5b between 2009 and 2012 [4]. Among countries and donors, a pledging conference for family planning in London in 2012 set targets for contraceptive use, creating the basis for the FP2020 reference group and secretariat. In addition, the UN has scaled up attention to child, early and forced marriage, passing a resolution in 2014 that put pressure on member-states to develop national legislation and protective policies [60].

As in the HIV/AIDS space, civil society groups have been central to the process of getting family planning and sexual and reproductive health on the global policy agenda. However, MNCH advocacy networks – while successful in positioning the Global Strategy as a key platform for consensus and commitment – continue to identify a mutually reinforcing set of technical and political barriers for greater action, including lack of funding, technical capacity, coordinating platforms, information flow, and inclusion in national planning and financing dialogues.

Power and participation is a particularly key issue. It was not until 2013, for example, that a civil society leader – Graca Machel, humanitarian and widow of Nelson Mandela – assumed the chair of PMNCH, even though NGOs account for two-thirds of the PMNCH membership by number [61]. In October 2015, youth and adolescent members of PMNCH finally succeeded in establishing their own constituency and seat on the board following a multi-year advocacy process. While youth are now often included on panels at global and regional events, they are often treated as symbols rather than experts, waiting their turn to speak as representatives of governments, donors and the UN take on more prominent roles. 

The question of how civil society groups claim and use power is beyond the scope of this paper. However, as a large constituency within MNCH advocacy networks, civil society movements are crucial to promoting social justice claims within national policy dialogue through the legitimacy and authenticity of the voices they represent. While stillborn babies, newborns, and small children cannot of course speak to their experiences, other community representatives, including adolescents, women, and parents, can provide powerful public testimony that transform how claims are heard.

In sum, the technical framing of causes, solutions and accountability within the global MNCH community, influenced by powerful scientific leaders and the MDG framework itself, has made it difficult for the network to fully benefit from its inherent claim on social justice. Community leaders, essential to expressing the interests and experiences of those most affected by MNCH policies, need greater support to play this political role and to engage in technical discourse.

As the experience of the HIV community in the MDG era has shown [62], civil society groups have the capacity to bring rights-based claims to centre stage, converting even complex technical problems into broad-based social movements that attract political attention.

## Conclusion

This paper applies the Shiffman and Smith framework on political attention to guide a discussion on the dynamic relationship between actor-networks, framing, and political context. This case study on the MNCH policy community concludes that global development frameworks have exerted significant pressure on ideational processes and framing, as well as network structures, behaviours and leadership, catalysing the alignment and realignment of both frames and networks to achieve and sustain influence.

This illustrates the challenges and opportunities of shifting political contexts. On the one hand, the shift from the MDGs to the post-2015 SDGs have presented the MNCH network with a vast action agenda. These include the urgent need to strengthen the links between MNCH and adolescent and reproductive health; promoting a stronger relationship between MNCH and infectious disease and non-communicable diseases; and in recognizing MNCH and public health more broadly as a product of social, economic and environmental determinants. This shift challenges network identity, membership and underlying conceptual concepts that have facilitated successful alignment and network coherence during the MDG era.

To date, networks have been opportunistic in embracing these changes. Changes been framed by network leaders as opportunities to assert rights-based narratives that accord with SDG norms as well as network core values. In this vein, network leaders have embraced youth groups to provide authentic leadership in response to the SDG focus on reproductive and adolescent health concerns, contributing network expansion and ambition.

While these recent changes have been adopted with little friction to date, network stability may be threatened if the SDG agenda narrows in practice, and choices in political attention begin to be made. At the same time, network structures that revolve around global secretariats in New York or Geneva must shift substantially if a truly country-led approach to planning, financing, advocacy and accountability is to be realised.

Therein lies important questions for the future: How will the lengthy SDG agenda be interpreted in practice by political leaders? Will “MDG era” concerns be sustained, or will attention shift to newer frames and networks, provoking a struggle for power and resources within this joined-up reproductive, maternal, newborn, child and adolescent health community focusing on national leadership? The strategic alliances between sub-communities forged in a more technocratic and apolitical MDG era may come under strain in an era focused on elevating concerns about equity and human rights. Indeed, the continuum of care concept -- the “conceptual glue” between RMNCH + A sub-communities -- may itself lose value in the context of a comprehensive SDG health goal that promotes a life course approach, moving beyond the mortality concerns of the MDGs: “Ensure healthy lives and promote well-being for all at all ages”.

Such struggles may not be contained within the women’s and children’s health community. The history of implementing the Alma Ata primary health care approach tells us that, when comprehensive agendas are seen as too ambitious or expensive to implement, they are subject to re-interpretation and narrowing through counter-movements, regardless of the formal consensus process that legitimised their creation. Whether that political struggle will occur, to be led by whom, is yet to be seen in relation to the broader SDG agenda, where health is now one of 17 inter-related goals.

In conclusion, this case of the MNCH policy community finds that idea-framing, actor behaviours and network development are highly interdependent processes, with political context exerting a significant impact on all such constructions. However, context alone cannot govern outcomes – the agency of networks to determine and sustain success through strategic realignments and reframing remains key.

Policy communities and their ideas are neither static nor impervious to change. The current transition between global development frameworks creates new space for advocacy networks to re-imagine and re-invent shared ideas that structure membership, leadership, and behaviours. At the same time, risk is inherent to change, presenting challenges to network coherence and cohesion and limiting overarching efforts to maintain political attention. When resources and attention are seen to shift too far to one side, policy sub-communities may retreat to their core values and disparate interests compete for dominance.

Can the MNCH/RMNCH + A community sustain the success of the past 10 years? Can techno-managerial discourses adapt to a more rights-based environment, in which the concept of accountability extends beyond quantitative measures to a truly social and political process of inclusive development and participation? In this sense, ideas and the framing of those ideas are part of a discourse within networks, which use their power and the surrounding political contexts to introduce shifts in policy, such as more political attention for women’s and children’s health.

The opportunities of change lie in adaptation, partnership and reinvention. These are the challenges that the women’s, children’s and adolescents’ health community will face during its ongoing ambition to bring health and well-being to the centre of the post-2015 framework.

## Footnotes

1 French [63] provides proposes a contrary explanation for improvements in child mortality during the Millennium Development Goal (MDG) era, attributing such reductions to coincidental economic growth during this time rather than the focusing power of global policy frameworks or even national public health expenditure. Critics, however, could rebut that while income is clearly a key determinant of household purchasing power of such things as nutritious food, mosquito nets and medicine, a confounding factor is that rising GDP often brings greater formal employment opportunities for women, which can exert a negative effect on mothers’ capacity to invest time in activities with proven child health benefits, such as antenatal and postnatal visits, breastfeeding, cooking healthy meals, collecting clean water and so on. As Murray [64] notes, the significant rise in Development Assistance for Health from USD11.6b in 2000 to USD33.1b in 2012 can be correlated with a strong emphasis on health in the MDGs, which itself had “broad societal appeal” (p.1391) and the capacity of influencing household investments.

2 “Political attention” is defined by Hafner and Shiffman [48] as occurring when “leaders of organisations express concern about issues publicly and privately, and when they back up this concern by allocating resources.”
